# Selective improvement of pulmonary arterial hypertension with a dual ET_A_/ET_B_ receptors antagonist in the apolipoprotein E^−/−^ model of PAH and atherosclerosis

**DOI:** 10.1177/2045893217752328

**Published:** 2017-12-20

**Authors:** Lewis Renshall, Nadine Arnold, Laura West, Adam Braithwaite, Josephine Pickworth, Rachel Walker, Mabruka Alfaidi, Janet Chamberlain, Helen Casbolt, A.A. Roger Thompson, Cathy Holt, Marc Iglarz, Sheila Francis, Allan Lawrie

**Affiliations:** 1 152607 Department of Infection, Immunity and Cardiovascular Science, University of Sheffield, Sheffield, UK; 2 159080 Institute for Cardiovascular Science, University of Manchester, Manchester, UK; 3 17431 Actelion Pharmaceuticals Ltd., Allschwil, Switzerland

**Keywords:** endothelin, macitentan, ApoE, pulmonary hypertension, atherosclerosis

## Abstract

Idiopathic pulmonary arterial hypertension (IPAH) is increasingly diagnosed in elderly patients who also have an increased risk of co-morbid atherosclerosis. Apolipoprotein E-deficient (ApoE^−/−^) mice develop atherosclerosis with severe PAH when fed a high-fat diet (HFD) and have increased levels of endothelin (ET)-1. ET-1 receptor antagonists (ERAs) are used for the treatment of PAH but less is known about whether ERAs are beneficial in atherosclerosis. We therefore examined whether treatment of HFD-ApoE^−/−^ mice with macitentan, a dual ET_A_/ET_B_ receptor antagonist, would have any effect on both atherosclerosis and PAH. ApoE^−/−^ mice were fed chow or HFD for eight weeks. After four weeks of HFD, mice were randomized to a four-week treatment of macitentan by food (30 mg/kg/day dual ET_A_/ET_B_ antagonist), or placebo groups. Echocardiography and closed-chest right heart catheterization were used to determine PAH phenotype and serum samples were collected for cytokine analysis. Thoracic aortas were harvested to assess vascular reactivity using wire myography, and histological analyses were performed on the brachiocephalic artery and aortic root to assess atherosclerotic burden. Macitentan treatment of HFD-fed ApoE^−/−^ mice was associated with a beneficial effect on the PAH phenotype and led to an increase in endothelial-dependent relaxation in thoracic aortae. Macitentan treatment was also associated with a significant reduction in interleukin 6 (IL-6) concentration but there was no significant effect on atherosclerotic burden. Dual blockade of ET_A_/ET_B_ receptors improves endothelial function and improves experimental PAH but had no significant effect on atherosclerosis.

## Introduction

Idiopathic pulmonary arterial hypertension (IPAH) is a devastating life-shortening condition.^1^ Although often thought to predominantly affect young women, recent data from the European COMPERA study highlights the increasing number of elderly patients aged >65 years diagnosed with IPAH.^2^ Atherosclerosis and atherosclerotic-associated diseases are the leading cause of cardiovascular disease and death worldwide.^3,4^ There are multiple risk factors for atherosclerosis, including genetics, high levels of C-reactive protein, and diet, but the impact of more traditional risk factors such as high levels of triglyceride and LDL cholesterol, smoking, and age still remains significant.^5,6^ Pro-inflammatory cytokines, such as those from the interleukin (IL) family, are upregulated in both atherosclerosis^7–9^ and PAH.^10,11^ Specifically, overexpression of IL-6 in mice can lead to spontaneous PAH and exacerbates chronic hypoxia-induced PAH^12^ and high IL-6 concentrations are also associated with increased mortality in patients with coronary artery disease and PAH.^7,11,13^ Interestingly, IL-1 (sitting upstream of IL-6) is also elevated in both humans and animal models of PAH and atherosclerosis, and inhibition of IL-1 has shown therapeutic potential;^8,10,14,15^ indeed data from our own lab demonstrated therapeutic efficacy of IL-1 receptor antagonist treatment on the high-fat diet (HFD)-fed apolipoprotein E-deficient (ApoE^−/−^) model of PAH.^16^

Mechanistically, endothelial dysfunction is evident in both PAH and atherosclerosis in the form of impaired endothelium-dependent and -independent vasorelaxation.^17–19^ Endothelin (ET)-1 is a potent vasoconstrictor formed by the conversion of Big ET-1 to ET-1 by ET-converting enzymes.^20^ ET-1 production and secretion can be regulated by inflammatory cytokines,^21,22^ hypoxia, and glucose,^23–25^ and is increased in the vessel wall of experimental models^26^ and human atherosclerotic lesions^26,27^ and PAH.^28^

The biological function of ET-1 can be reduced through the administration of endothelin receptor antagonists (ERAs). ERAs bind to either one or both ET_A_/ET_B_ receptors on smooth muscle cells (SMC) or endothelial cells and reduce the vasoconstriction associated with ET-1.^29–31^ ERAs improve the prognosis of World Health Organization (WHO) functional class II and III PAH patients^32^ and ET_A_-selective or dual ET_A_/ET_B_ antagonism were shown to increase endothelial function and improve forearm^33,34^ and coronary artery^35^ blood flow in patients with atherosclerosis.

Since atherosclerosis and PAH have these shared pathophysiological processes, and that ET-1 stimulates production and secretion of IL-6 in human vascular SMCs via nuclear factor-κB (NF-κB),^21^ and with evidence of an aging population of IPAH patients, we sought to further investigate the potential impact of macitentan treatment on the Paigen HFD-fed ApoE^−/−^ model of concomitant atherosclerosis and PAH.^15,16^ We hypothesized that treatment with a dual ERA would improve endothelial function, reduce inflammatory cytokines, improve cardiac hemodynamics, and thus alleviate atherosclerotic lesion burden.

## Methods

### Animals

ApoE^−/−^ mice were obtained from Jackson Laboratories (Bar Harbor, ME, USA) and were on a C57BL/6 J background. Male mice weighing 22–30 g at the age of 12 weeks were fed a Paigen HFD (18.5% fat, 0.9% cholesterol, 0.5% cholate, and 0.2959% sodium) or standard chow (4.3% fat, 0.02% cholesterol, and 0.28% sodium). Diets were supplied by Special Diet Services, Braintree, UK. At the age of 16 weeks, mice fed a Paigen HFD were randomized to either intervention (ApoE^−/−^ HFD macitentan; 30 mg/kg/day) or treatment-naïve group (ApoE^−/−^ HFD) for a further four weeks (n = 10/group). Mice fed a standard diet were treatment-naïve (ApoE^−/−^ chow). All procedures conformed to UK Home Office Regulations (Animals Scientific Procedures Act 1986; Project license 40/3517) and were approved by the University of Sheffield Project Review Committee.

### Echocardiography

At week 20, transthoracic echocardiography was performed (n = 8/group) using the RMV707B scan head on the Vevo 770 system (VisualSonics, Toronto, ON, Canada). There was a single operator blinded to the intervention status of the mice. Mice were placed on a heated platform with rectal temperature, heart rate, and respiration rate monitored continuously. Anesthesia was induced and maintained using isoflurane through oxygen. Right ventricular (RV) free wall and left ventricular (LV) parameters were obtained as previously described.^36^ Cardiac output was derived by measurement of the flow and diameter at the junction between aortic outflow tract and aortic valve. Cardiac index was normalized by body weight and all measurements were taken at non-inspiratory phase of the cardiac cycle. Analysis was performed using the Vevo 770 V3.0 VisualSonics software.

### Cardiac catheterization

Immediately after echocardiography, animals were maintained under 1–2% isoflurane while performing right and LV catheterization via either the right internal carotid artery or the right external jugular vein. Pressure-volume (PV) data were collected by Millar ultra-miniature catheters (PVR-1045; mouse LV, PVR-1030; mouse RV: Millar Instruments, Inc., Houston, TX, USA) coupled to a Millar MPVS 300. Data were acquired using Powerlab 8/30 (AD Instruments, Oxfordshire, UK) and recorded using LabChart 7 Pro software (AD Instruments). PVAN v2.3 (Millar Instruments, Inc.) was used to analyze PV data as previously described.^36^

### Assessment of RV hypertrophy

Following cardiac catheterization, animals were euthanized by cervical dislocation under 2% isoflurane. Cardiac tissue was collected and fixed in 10% neutral buffered formalin for 24 h and washed with PBS. Using a modified Fulton index,^37^ the weight of the right ventricle free wall relative to the left ventricle and septum was used to determine an estimate of RV hypertrophy (n = 7/group).

### Endothelial function of thoracic aortae

*Mounting.* Thoracic aortas from HFD-fed mice were harvested to assess vascular reactivity using wire myography (ApoE^−/−^ HFD macitentan n = 4; ApoE^−/−^ HFD n = 4 mice). Aortae were mounted onto a Danish Myo Technologies 610 M wire myograph (DMT, Denmark), warmed to 37℃, and gassed with 95% air/5% CO_2_/balance N_2_ for 30 min in physiological salt solution (PSS in mM; 119 NaCl, 25 NaHCO_3_, 4.7 KCl, 1.17 MgSO_4_, 1.6 CaCl_2_, 1.17 KH_2_PO_4_, 5.5 glucose, 0.03 EDTA [free acid] at pH 7.4).

*Normalization.* Incremental stretch was applied to each vessel using the micrometer attached to the myograph chamber and passive tension was recorded for 30 min until a passive steady resting tension of 5 mN was achieved.

*Experimental procedure.* Post-equilibration, aortas were exposed to two separate washes with depolarizing solution (KPSS in mM: 63.7 NaCl, 25 NaHCO_3_, 60 KCl, 1.17 MgSO_4_, 1.6 CaCl_2_, 1.17 KH_2_PO_4_, 5.5 glucose, 0.03 EDTA [free acid] at pH 7.4). After washing with PSS, aortas were pre-contracted with 10 µM phenylephrine and exposed to ACh (1 × 10^−9^ – 3 × 10^−5 ^M). After washing with PSS, vessels were pre-contracted with 10 µM phenylephrine and exposed to the nitric oxide donor sodium nitroprusside (SNP; 1 × 10^−9^ – 3 × 10^−5 ^M).

### Immunohistochemistry

The left lung was perfusion fixed via the trachea as previously described.^32^ Briefly, the left lung was inflated with 10% neutral buffered formalin at 20 cm of H_2_O. Lungs were processed in paraffin wax and sectioned (5 µm). Sections were histologically stained with Alcian Blue Elastin van Gieson (ABEVG). For immunohistochemistry, sections were stained with α-smooth muscle actin (SMA; M0851; Dako, Cambridgeshire, UK) for SMC identification and von Willebrand factor (vWf; A0082; Dako) to identify endothelial cells. Standard immunohistochemical techniques were applied, and both IgG and no primary antibody negative controls were used. The Axiocam 506 Color System (Zeiss) was used for microscopy and analysis was performed in Zen 2 Blue Edition (Zeiss).

### Quantification of pulmonary vascular remodeling

The degree of pulmonary muscularization was assessed and analyzed as a percentage of total non-muscularized vessels.^36^ Vessels were categorized into two groups based on their size: small arterioles (<50 µm in diameter) and medium pulmonary arteries (>51–100 µm).

### Assessment of atherosclerotic lesion burden

Aortic sinus paraffin-embedded sections were stained with ABEVG to assess mean lesion to cross-sectional area ratio and martius scarlet blue to assess collagen content. Sections were also stained immunohistochemically with α-SMA, as described above. Whole mount aortae, which included aortic arch and descending aorta, were stained with Oil Red O (Sigma, UK) to assess lesion area as a percentage of total aortic area. Scoring was performed in a blinded manner.

### Enzyme linked immunosorbent assays

Before sacrifice, a cardiac puncture was performed and serum samples were collected for cytokine analysis (n = 4–9/group). To assess concentration of soluble cytokines, the Cytometric Bead Assay Flex sets (BD Biosciences, Oxford, UK) for insulin, IL-1β, IL-6, IL-10, hepatocyte growth factor (HGF), tumor necrosis factor (TNF)-α, monocyte chemoattractant protein 1 (MCP-1), Leptin, interferon-gamma-inducible protein-10 (IP-10), stromal cell-derived factor (SDF)-1 a, and C-reactive protein (CRP) were used.

### Statistical analyses

When comparing two groups, a Mann–Whitney *U* test was used for statistical analyses. When comparing the medians of three groups or more, a Kruskal–Wallis test followed by Dunns post-hoc test (95% confidence interval) was undertaken. For myography data in thoracic aortae, two-way analysis of variance was performed with Bonferroni post-hoc test. *P* < 0.05 was deemed to be significant (Prism 6.0; Graphpad Software). Data are presented as mean ± SEM, unless otherwise stated. For technical reasons, certain procedures were unable to be completed on each individual animal and thus there is variation in the number of data points. An inability to quantify pulmonary artery flow using echocardiography, inability to catheterize left ventricle, and a serum sample out of detection range of standard curve were the most common reasons for missing data.

## Results

**Fig. 1.**
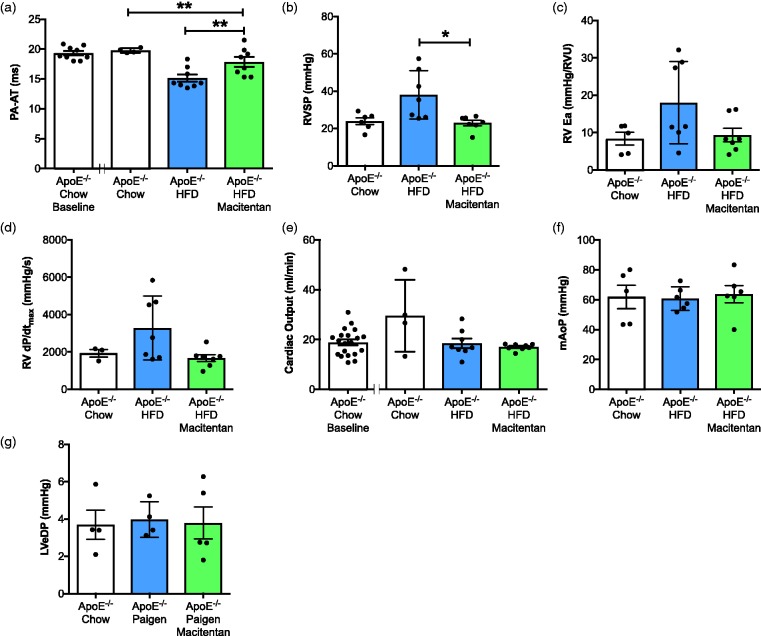
Macitentan treatment reduces hemodynamic indices of PAH in HFD-fed ApoE^−/−^ mice. Bar graphs show (a) pulmonary artery acceleration time (PA-AT), (b) RV systolic pressure (RVSP), (c) RV arterial elastane (RVEa), (d) RV dP/dt_max_ in mm Hg/s, (e) cardiac output, (f) mean aortic pressure (mAoP), (g) LV end-diastolic pressure (LVeDP). (a, f) Measures were made using Vevo 770 echocardiography v3.0. All other measures were made using closed chest cardiac pressure volume catheterization. Bars represent mean ± SEM, n = 6–20, individual animals represented by dots. (a–g) Statistical differences between chow-fed ApoE^−/−^, HFD-fed, and HFD-fed + macitentan were assessed by Kruskal–Wallis analysis of variance with Dunn’s multiple comparison post-hoc test; *P < 0.05, **P < 0.01.

*Macitentan improves cardiac hemodynamics associated with PAH phenotype in HFD-fed ApoE^−/−^ mice.* HFD-fed ApoE^−/−^ mice developed a PAH phenotype similar to that previously described^16,36^ as assessed by a decrease in pulmonary artery acceleration time (PA-AT; Fig. 1a), increase in RV systolic pressure (RVSP; Fig. 1b), and changes in RV function (Fig. 1c, d). Treatment with macitentan significantly increased PA-AT (Fig. 1a), reduced RVSP (23 mmHg vs. 38 mmHg; Fig. 1b) and improved RV function (Fig. 1c, d). There was no effect of macitentan on LV cardiac output (Fig. 1e), mean aortic blood pressure (Fig. 1f), or LV end-diastolic pressure (LVeDP; Fig. 1g). As previously reported,^16,36^ there was no significant increase in RV hypertrophy (RVH) in HFD-fed ApoE^−/−^ mice, and there were no significant differences in macitentan-treated HFD-fed ApoE^−/−^ mice when compared with treatment-naïve ApoE^−/−^ mice (data not shown).

**Fig. 2.**
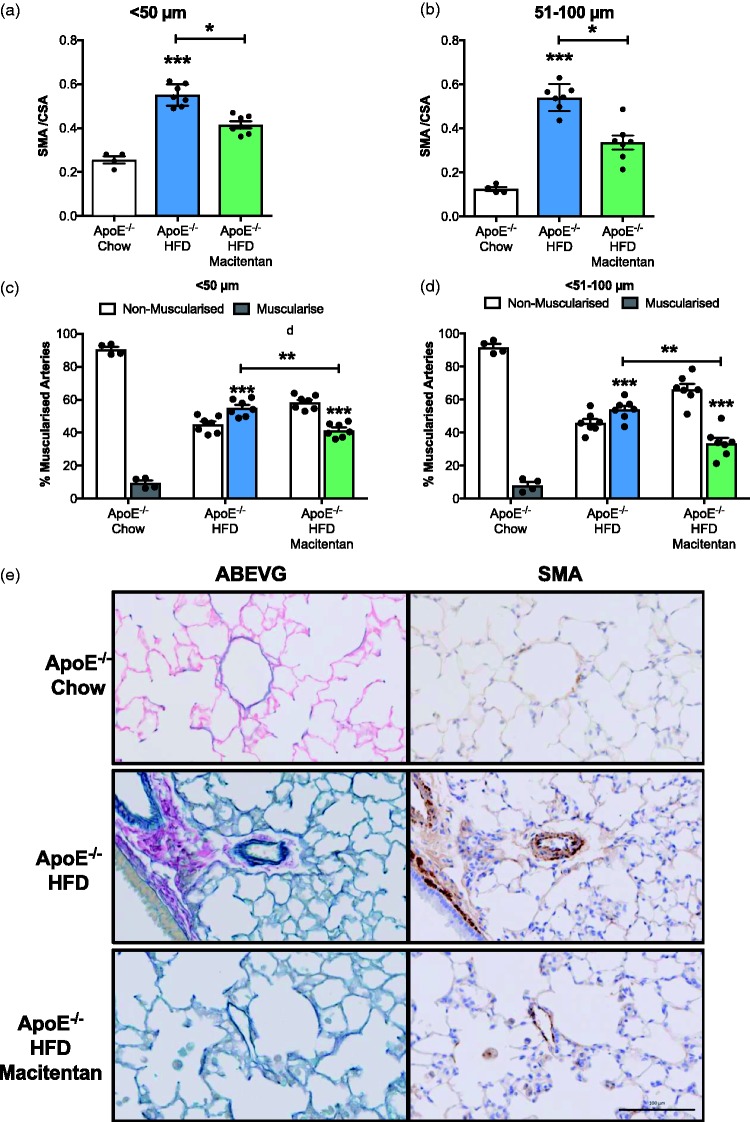
Macitentan treatment reduces pulmonary vascular remodeling in HFD-fed ApoE^−/−^ mice. Bar charts (a, b) illustrating the degree of medial wall thickening (identified by SMA) as a ratio of total vessel size (SMA / CSA) in <50 µm arteriole (a) and small to medium pulmonary arteries (b). (c, d) The percentage of muscularized and non-muscularized arterioles (c) and arteries (d). Representative images of ABEVG and SMA-stained tissue (e); ABEVG and SMA-stained images of pulmonary arteries from ApoE^−/−^ mice that were fed either chow (top), HFD with no treatment (middle), and HFD with macitentan (bottom). Scale bar represents 100 µm. (a, b) Bars represent mean ± SEM, n = 7. Kruskal–Wallis analysis of variance; *P < 0.05, ***P < 0. (c, d) Stars represent level of statistical significance when compared with chow-fed ApoE^−/−^ mice; ****P < 0.0001. Post-hoc analysis shows statistical differences between macitentan-treated and treatment-naïve HFD-fed mice (*P* < 0.0001).

*Macitentan reverses pulmonary vascular remodeling associated with the PAH phenotype in HFD-fed ApoE^−/−^ mice.* Histologic and immunohistochemistry analysis of serial lung tissue sections revealed significant pulmonary vasculopathy consistent with a PAH phenotype in HFD-fed ApoE^−/−^ mice. Analysis of media/CSA ratio in small resistance pulmonary arterioles <50 µm in diameter (Fig. 2a) and the small to medium 51–100 µm pulmonary arteries (Fig. 2b) demonstrated significant muscularization in treatment-naïve HFD-fed ApoE^−/−^ animals when compared to standard chow-fed ApoE^−/−^ mice. Treatment with macitentan resulted in a significant decrease in the media/CSA for both the small pulmonary arterioles (<50 µm; Fig. 2a and e), and the small to medium pulmonary arteries (51–100 µm; Fig. 2b). Similarly, there was a significant increase in the percentage of muscularized arterioles (<50 µm; Fig. 2c and e) and arteries (51–100 µm; Fig. 2d) in HFD-fed ApoE^−/−^ mice that was also reduced by treatment with macitentan.

**Fig. 3.**
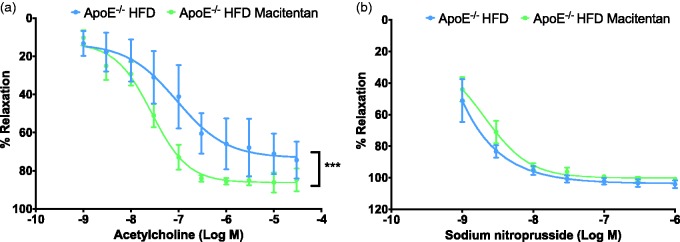
Macitentan treatment improves vascular function in thoracic aortae. Graphs show cumulative dose response curves to increasing concentrations of acetylcholine (a) or sodium nitroprusside (b) in thoracic aortae pre-contracted with 10 µM phenylephrine. Data are expressed as mean ± SEM and presented as a % of endothelium-dependent (a) or -independent (b) relaxation, n = 4. *P < 0.05, ***P < 0.001. Statistical differences between treated and untreated HFD-fed mice were assessed by two-way analysis of variance with Bonferroni post-hoc analysis.

*Macitentan improves endothelial-dependent vascular function associated with atherosclerotic phenotype in HFD-fed ApoE^−/−^ mice.* In thoracic aortae explanted from macitentan-treated HFD-fed ApoE^−/−^ mice, endothelial-dependent relaxation of pre-contracted thoracic aortas was markedly increased compared to untreated HFD-fed ApoE^−/−^ mice (Fig. 3a). Conversely, when thoracic aortas were once again pre-contracted with phenylephrine, there was no significant effect of sodium nitroprusside-mediated endothelial-independent relaxation in macitentan-treated animals compared with treatment-naïve littermates (Fig. 3b). There were no differences in maximal contraction to 60 mM high-potassium depolarizing salt-solution or 10 µM phenylephrine between treatment-naïve and macitentan-treated ApoE^−/−^ littermates (data not shown).

**Fig. 4.**
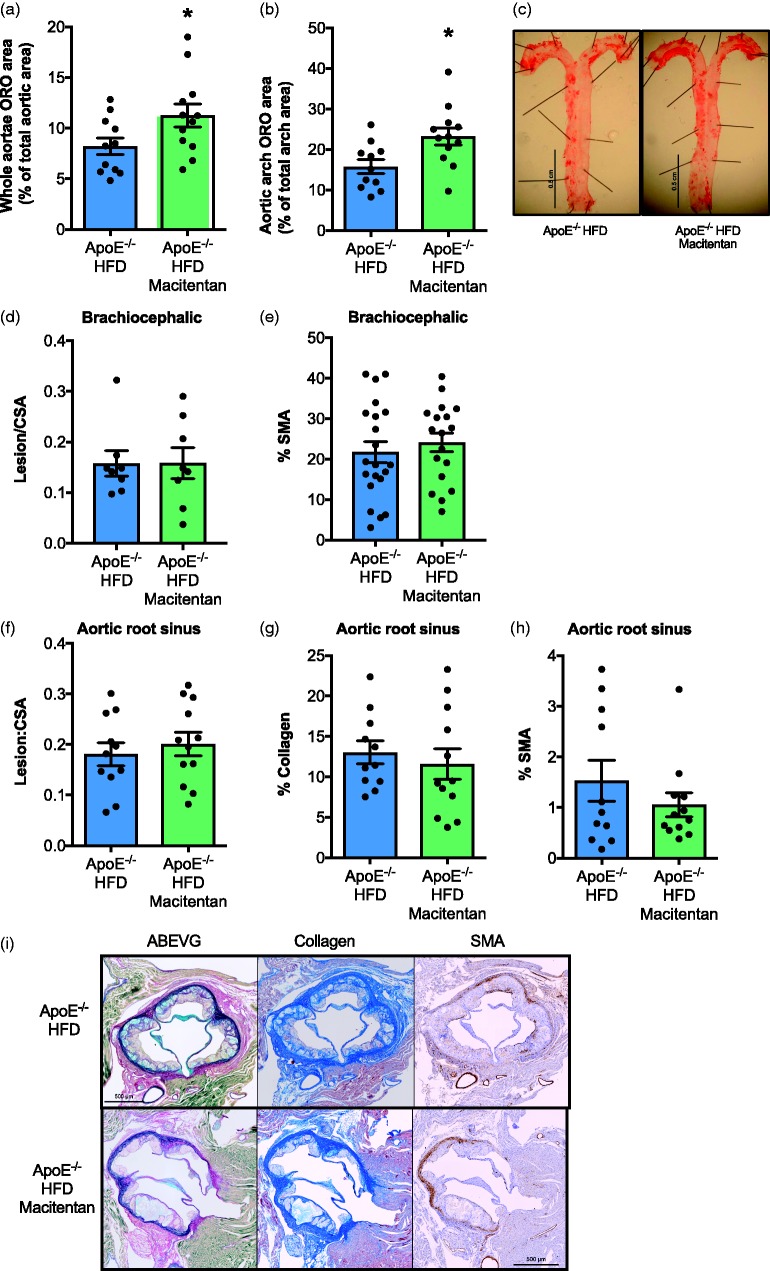
Macitentan treatment does not alter the composition or atherosclerotic burden in HFD-fed ApoE^−/−^ mice. Bar charts show ORO stain as a % of total aortic area (a), and within the aortic arch as a % of total arch area (b). (c) Representative images of ORO in control and macitentan-treated mice. (d) Bar charts show the ratio of atherosclerotic lesion area within the brachiocephalic artery normalized to cross-sectional area, and (d) the % α-SMA positive tissue within the brachiocephalic sections. (f) The ratio of lesion area to cross-sectional area within the aortic root sinus, (g) % collagen content, and (h) % α-SMA positive tissue in aortic root sections. (i) Representative aortic root histological sections from control and macitentan-treated HFD-fed ApoE^−/−^ mice stained with ABEVG, martius scarlet blue (collagen), and α-SMA. Bars represent mean ± SEM. Statistical differences between sections from treated and untreated mice were assessed by Mann–Whitney *U* test, n = 11–12. Individual mice are represented by dots. *P < 0.05. Statistical differences between groups were measured by Mann–Whitney *U* test.

*Macitentan treatment had no effect on atheroma lesion burden in HFD-fed ApoE^−/−^ mice.* On examination of explanted whole aortae stained *en face* with Oil Red O (ORO), there appeared to be a small but significant increase in lipid deposition in macitentan-treated animals (Fig. 4a and c). This appeared to be due to greater accumulation of lipid specific to the aortic arch region (Fig. 4b) and not the descending aorta (data not shown). However, immunohistological analysis of both the brachiocephalic artery (Fig. 4d and e) and aortic root sinus (Fig. 4f–i) demonstrated that there was no significant effect of macitentan treatment on atherosclerotic lesion burden or composition. Macitentan treatment did not alter the size of the lesion, as assessed by the ratio of lesion to cross-sectional area (Fig. 4d), or the content of SMA in the lesion of the brachiocephalic artery (Fig. 4e) compared with sections from control mice. Further histological assessment of the aortic root sinus also showed no significant change in the ratio of lesion to cross-sectional area (Fig. 4f and i), collagen content (Fig. 4g and i) or vascular SMC content (Fig. 4h and i) of macitentan-treated mice compared with treatment-naïve controls.

**Fig. 5.**
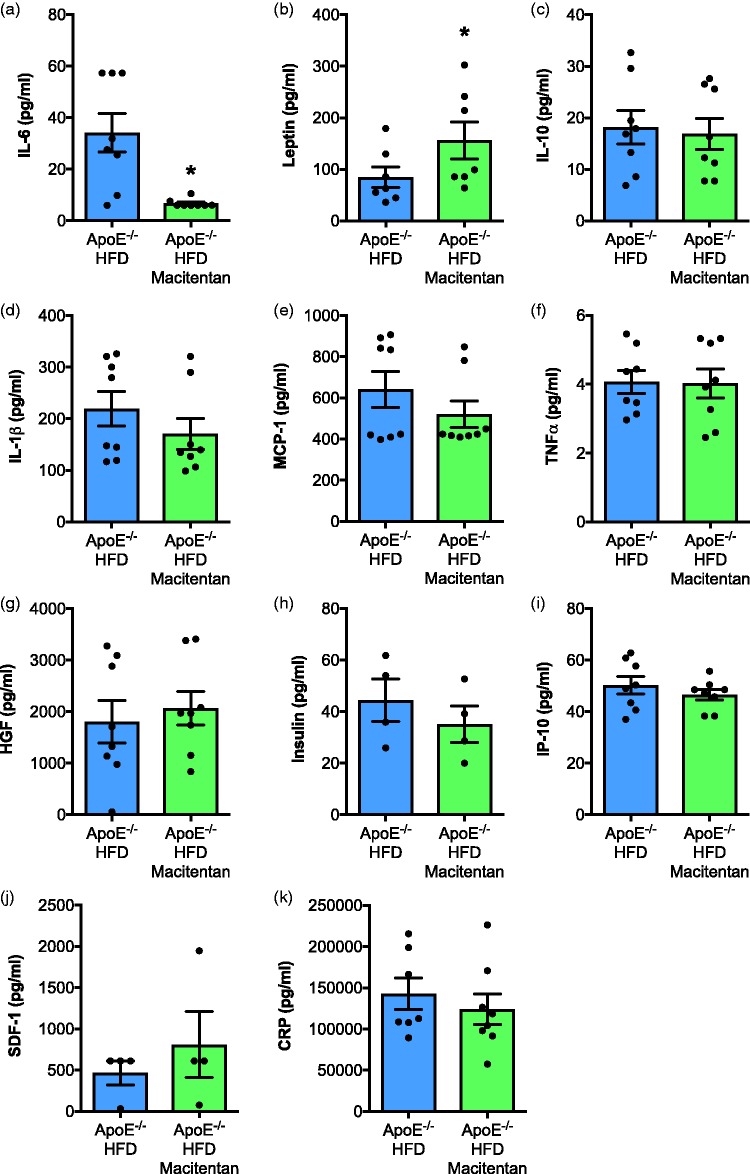
Macitentan treatment induces changes in inflammatory plasma cytokines in HFD-fed ApoE^−/−^ mice. Bar charts show the plasma concentration of (a) IL-6, (b) leptin, (c) IL-10, (d) IL-1β, (e) MCP-1, (f) TNF-α, (g) HGF, (h) insulin, (i) IP-10, (j) SDF-1, and (k) CRP. Bar graphs represent mean ± SEM, n = 4–9 with individual animals represented by dots. *P < 0.05. Statistical differences were assessed by Mann–Whitney *U* test.

*Macitentan decreases IL-6 levels in HFD-fed ApoE^−/−^ mice.* Treatment with macitentan resulted in a significant reduction in circulating levels of IL-6 (Fig. 5a) and increased the concentration of leptin (Fig. 5b) in serum. There were no significant differences in TNF-α, MCP-1, IL-1β, IL-10, IP-10, SDF-1, CRP, insulin, or HGF (Fig. 5c–k).

## Discussion

To our knowledge, this is the first time macitentan has attenuated established PAH in mice. In a novel model of concomitant PAH and atherosclerosis, we found a significant reduction in muscularization of small vessels and associated improvements in hemodynamics. Our results are consistent with pre-clinical studies using other models of pulmonary hypertension^38^ and with clinical studies in PAH patients.^32^ Macitentan treatment increased endothelium-dependent dilatation but was not associated with a reduction in atherosclerosis lesion burden at the aortic root or the brachiocephalic artery.

The full molecular mechanisms by which ERAs attenuate PAH are still not fully understood, although there is clear evidence for vasodilatory effects that may result in flow-mediated improvements in remodeling. However, we also found a significant reduction in circulating IL-6 in macitentan-treated Paigen diet-fed ApoE^−/−^ mice. Pre-clinical^12^ and clinical^10,11^ studies identify IL-6 as a potential key regulator of PAH pathogenesis and therefore a putative drug target. Indeed, the direct effect of targeting IL-6 in PAH will soon become clearer with the conclusion of the TRANSFORM-UK study (NCT02676947). Studies by McMillen have previously demonstrated that ET-1 can stimulate IL-6 production from human monocytes.^39^ We propose that the same is occurring in this model, driving increased IL-6 in the Paigen diet-fed ApoE^−/−^ mice, and that levels fall following the addition of macitentan. Previous studies have shown that administration of an IL-6 R antagonist in patients at high risk of coronary artery disease led to a reduction in IL-6, an increase in endothelial function, induction of dyslipidemia, and an increase in total lipid plasma content.^40^ There is a suggestion that the dyslipidemia as a result of reduced IL-6 may in fact be causing the lipid profile to alter to a more anti-inflammatory composition.^41^ In our model, quantitative assessment of aortic sinus and brachiocephalic lesion burden suggested there were no differences in lesion size or content between treatment-naïve and macitentan-treated HFD-fed ApoE^−/−^ mice. ORO staining of the uppermost surface of the aortic tree dissected from experimental animals suggested increased lipid rich areas in the treated mice. However, as this technique can be highly influenced by tissue preparation, and since the much more robust and accepted histological assessment of aortic root and the brachiocephalic artery did not show any significant difference, we conclude that macitentan does not adversely affect experimental atherosclerosis in our joint model of PAH and atherosclerosis. Our data contrast with other animal studies demonstrating that either dual ET_A_/ET_B_ or selective ET_A_ receptor antagonists reduce the development and progression of atherosclerosis in both Western diet-fed ApoE^−/−^ and Ldlr^-/–^deficient mice.^42–45^ . We speculate that there may be two explanations for a lack of effect of macitentan on atherosclerotic lesion burden in our study; the length of treatment may not have been sufficient and/or atherosclerotic lesion development and progression was more pronounced in our novel model compared with the traditional Western diet. However, consistent with studies assessing the effects of ERAs on clinical atherosclerosis,^30,34,35^ macitentan was able to increase endothelium-dependent relaxation of thoracic aortas.

Our study utilized an eight-week fat-feeding regimen commencing at the age of three months. While this is a similar age used in previous studies investigating the effect of ERAs on atherosclerosis,^42–45^ macitentan was only administered for the final four weeks of the model. In the context of atherosclerosis, there seems little consensus on the length of treatment of ERAs, with some studies opting for a 6–10-week treatment period,^42,44,45^ while others opt for a longer 30-week period.^43^ We chose a four-week treatment period as this is known to be beneficial in PAH,^16^ and as such we wanted to assess whether it could impact on atherosclerosis in our Paigen diet-fed model. Indeed, a recent study from Houde et al. showed that a six-week treatment of macitentan was sufficient to reduce aortic lesion development in ApoE^−/−^ mice after 17 weeks of a traditional Western diet.^42^ However, it must be noted that there is a far greater atherosclerotic lesion burden in ApoE^−/−^ mice fed a Paigen diet when compared with ApoE^−/−^ mice fed a traditional Western diet.^15^ Taken together, these data suggest either increasing the length of treatment or commencing treatment at an earlier stage in atherogenesis may reduce lesion burden or dampen the progression of atherosclerosis in our model. Indeed, clinical data suggest that ERAs are only able to reduce plaque progression in coronary arteries at the early stages of atherosclerosis and in the presence of endothelial dysfunction.^46^

Macitentan treatment convincingly led to increased endothelium-dependent relaxation in aortae and a reduction in the pro-inflammatory cytokine IL-6. Serum IL-1β was not altered, but this is unsurprising given the inherent difficulties in measuring this cytokine.^47^ In high-risk patients with PAH and concomitant atherosclerosis, worsening endothelial function and raised inflammatory markers such as CRP and IL-6 are quantifiable; thus, our data suggest macitentan may be of most benefit to these patients.

In summary, macitentan significantly reduced the main pathological features associated with PAH such as pulmonary vascular remodeling and cardiac dysfunction. Macitentan treatment was associated with increased endothelium-dependent relaxation in aortae, a surrogate marker of endothelial function. Using our model of advanced atherosclerosis, there was no effect of macitentan on atherosclerotic lesion burden and treatment did not alter the morphology or the stability of plaques within the aortic sinus or brachiocephalic artery. We therefore suggest that macitentan treatment is able to improve endothelial function and reduce inflammatory mediators such as IL-6, but this did not translate through to reduced atherosclerosis in our study setting. Given the numerous successes of ERAs in experimental and clinical atherosclerosis, combined with our results suggesting improvements in endothelial function and markers of inflammation, we propose future studies should assess the effects of the current generation of ERAs, such as macitentan, in clinical atherosclerosis.
